# Study-related behavior patterns of medical students compared to students of science, technology, engineering and mathematics (STEM): a three-year longitudinal study

**DOI:** 10.1186/s12909-019-1696-6

**Published:** 2019-07-15

**Authors:** Edgar Voltmer, Katrin Obst, Thomas Kötter

**Affiliations:** Institute for Social Medicine and Epidemiology, University of Lübeck, Ratzeburger Allee 160, 23562 Lübeck, Germany

**Keywords:** Students, medical, STEM students, Health behavior, Burnout, professional, Health promotion

## Abstract

**Background:**

Medical education is perceived as demanding and stressful. Whether this is particularly pronounced in this course of study remains under debate.

**Methods:**

We used the questionnaire “Work-Related Behavior and Experience Patterns (Arbeitsbezogene Verhaltens- und Erlebensmuster (AVEM))” to assess the development of study-related behavior and experience patterns in medical (*n* = 584) and STEM students (*n* = 757) at one German university, with a special emphasis on gender differences, over 3 years of study. Students were surveyed at the beginning of their studies (t0) and again in each consecutive summer semester (t1-t3). Both cross-sectional and longitudinal data were generated and analyzed. Results in the abstract are from the cross-sectional analysis.

**Results:**

Freshman medical students presented with a larger proportion of students with a healthy pattern (58.1%) than STEM students (42.5%). In both groups this proportion decreased to 33.8%/25.1% at t2, with only a minor improvement at t3 (38.1/27.0%). Correspondingly, the proportion of students with a burnout-related risk pattern increased from 8.0% (Med)/13.7% (STEM) to a maximum of 16.9% at t2 in medical students and 27.0% at t3 in STEM students. In both groups female students showed a more unfavorable distribution of patterns and a higher vulnerability, especially in the area of resistance toward stress.

**Conclusions:**

The unfavorable development of behavior and experience patterns in both student groups demonstrates increasing study related stress and emphasizes the need for prevention and health promotion on an individual and a contextual level.

## Background

Medical education is perceived as very demanding and stressful. A number of cross-sectional studies report stress, anxiety, depression, and burnout [1, 2] as possible consequences of high workload, frequent testing, strict absence rules, and increasing responsibility for patients [3, 4]. Results from three cross-sectional surveys at different points in medical education and work-life indicated that this might be an increasing phenomenon throughout the course of study [5]. Indeed, a number of longitudinal studies demonstrated that life satisfaction and healthy behavior and experience patterns decreased and symptoms of anxiety, depression or burnout increased during medical education [6–8].

### Stress development in medical and other students

Whether these developments are particularly pronounced in the course of medical education has not been thoroughly investigated to date. Comparisons with other student groups or age matched peers are few and the results are inconsistent. In a review [9], the majority of studies found a lower prevalence of depression in medical students compared to law students and other student groups [10, 11]. However, in some of the studies examined, no statistically significant difference was found [12, 13]. Medical students had a greater commitment to their chosen career [14] and spent significantly more hours in class, laboratories, on wards, or studying than law students [15]. Studies from US medical schools showed better health compared to age-matched college students at matriculation [16] but poorer psychosocial health in the course of study [2]. In a study of tertiary students of Medicine, Psychology, Law and Mechanical Engineering the proportion of distressed students was 4.4 times higher than in age matched peers. However, in this study, medical students had lower levels of distress compared to law or engineering students [17]. The medical education course in Germany was not reorganized according to the Bologna declaration, a process to harmonize the European higher education system, but it still takes 6 years in total with 5 years of study and a practical sixth year. It ends with the state examination and Approbation (license to practice medicine). At the University where we conducted the study, a classical curriculum is offered with two pre-clinical years followed by the first major examination (“Physikum”). Then after three clinical years the course of study is completed with a practical year in three clinical areas.

Science, technology, engineering and mathematics (STEM) students have not been thoroughly surveyed in terms of psychosocial issues and were therefore chosen for this study as a comparison group. The course of study in the STEM faculties is organized according to the recommendations of the Bologna Process. After 3 years of study, a bachelor’s degree can be obtained. After another 2 years, a master’s level degree is possible. After the implementation of Bachelor (BA) and Master (MA) programs in Germany, a decrease in flexibility, and an increase in workload and study-related stress has been described for the affected subjects (e.g., STEM subjects; [18]). However, again, results are inconsistent. In surveys of the Free University of Berlin 74% of the BA students complained about performance pressure [19] and 41% suffered severe emotional exhaustion correlated with study conditions [20]. However, an analysis of students who sought help from a counseling center found no major program-specific effects in BA or MA students [21, 22]. One of the rare studies that addresses stress in STEM students found increasing levels during the first year in two large universities in the United States [23].

According to the transactional model of stress and coping [24] demanding situations in study, work, or daily life are not stressors per se. Depending on an individuals’ subjective perception of the stressor and the perceived coping resources available the same situation could be experienced for example as a challenge or threat. An affected individual is no passive victim of circumstances but an active element in dealing with professional or study-related stressors [25]. The interplay between perceived stress and coping behavior is evaluated by the instrument “Work-Related Behavior and Experience Patterns (Arbeitsbezogene Verhaltens- und Erlebensmuster (AVEM))” that we used in this study. It identifies four work−/study-related patterns that are described as pattern G – “Health” (Muster G – “Gesundheit”), pattern S – “Unambitious” (Muster S – “Schonung/Schutz”), risk pattern A – Overexertion (Risikomuster A – “(Selbst-)Überforderung”; the label refers to the type A behavior described by Friedman and Rosenmann [26]) and risk pattern B – Burnout (Risikomuster B – “Burnout; s. method section).

### Gender differences in the experience of study-related stress

Studies in the general population regarding psychosocial strain and symptoms often report a higher vulnerability of females compared to males. Prevalence of major depression and anxiety for example were found consistently twice as high in women than in men [27–29]. In Germany about two thirds of freshman medical students are female [30]. The proportion of female students in STEM subjects is significantly lower but increasing [31]. It would be of interest – not least for the planning of health-promotion measures – if the perception and development of study-related strain appears to be different between genders. In most of the above-mentioned studies no gender-sensitive analysis was reported. Where they are, results are inconsistent but tend towards a higher vulnerability in female students. A recent study reported that depression, anxiety, somatization and prevalence of psychotropic substance use in medical students were more pronounced in female students [1]. A cross-sectional survey of fifth-year medical students also found a significant difference, with a lower proportion of a healthy pattern in female students [5]. However, in a longitudinal study from first to second year in medical students no significant difference was observed [32]. In first year medical students there was a stronger decrease in the subjective perception of general health in female students compared to their male counterparts [33].

In the present study, we sought to compare, in a longitudinal design, the development of study related behavior and experience patterns of medical and STEM students and the extent to which male and female students differed in this respect. We report the follow-up studies of three cohorts of medical and STEM students in their first 3 years of study. For STEM students who complete their program in the designated period of time this means up to the end of their program. Based on the aforementioned studies we hypothesized that 1) students’ perception of stress increases in the course of study, which would be seen in more unfavorable behavior and experience patterns. Despite conflicting results about the question of whether students’ stress is particularly pronounced in medical education we hypothesized that 2) the pattern distribution would present higher proportions with risk patterns in medical compared to STEM students and there would be a greater increase of risk patterns and decrease of the healthy pattern in medical than in STEM students. Given the tendency to higher vulnerability in women regarding psychosocial stress, we hypothesized that 3) this development may be more prominent in female students than in their male counterparts.

## Methods

### Study design and setting

Data was drawn from an ongoing prospective, longitudinal observational study at the University of Lübeck, a public university with a focus on medicine and life sciences [33, 34]. From 2011 we invited all Medicine and STEM freshmen groups at the University of Lübeck to participate. The baseline surveys (t0) were taken in class during the pre-course week (prior to the beginning of courses). The follow-up surveys (t1-t3) were taken online in June during the respective summer semesters. There were no exclusion criteria. A 5 € book voucher per completed questionnaire was used as an incentive for participants. For this study, three cohorts of medical and STEM students in their first 3 years of study were analysed (Freshmen in 2011, 2012 and 2013 were followed through to 2014, 2015, and 2016).

### Instrument

We report here the results of the short form of the standard instrument: “Work-Related Behavior and Experience Pattern (AVEM)” [35].

The AVEM was developed to examine personal experiences with work-related stress and typical coping behaviors. The instrument comprises 11 separate scales that cover the following three major domains: 1) professional commitment (subjective significance of work, career ambition, tendency to exert, striving for perfection, emotional distancing), 2) resistance towards stress (resignation tendencies, offensive (i.e. proactive) coping with problems, balance and mental stability), and 3) emotional well-being (in the context of work; satisfaction with work, satisfaction with life, experience of social support). We used the short form of the AVEM in a student adapted version, which comprises 44 items scored on a 5 point Likert scale where 1 represents strongly disagree and 5 represents strongly agree (for item examples see [32]). From a cluster analysis of the initial AVEM sample group, comprised of 1,598 representatives from different professions four different types of work-related experience and behavior patterns were identified and could be described as follows [35]:

#### Pattern G: “health”

The healthy pattern G is characterized by a good balance between the domains of professional commitment, resistance towards stress and emotional well-being. Participants with this pattern are ambitious at work but also cope well with stress. They score high in the dimensions of satisfaction with work and life and experience of social support.

#### Pattern S: “unambitious”

The most prominent impression of this pattern are the lower scores in the dimensions of professional commitment and high scores in emotional distancing from work. Longitudinal data prove that this could either be due to a relaxed attitude that does not take work very seriously and that seeks and finds satisfaction in activities outside work. On the other hand the lack of professional commitment and the detachment from work could also be an early sign of demotivation and frustration that may later lead to burnout.

In contrast to these first two patterns the next two are termed as risk patterns, as they have been repeatedly shown to be correlated to symptoms of illness and poor health [36, 37].

#### Risk-pattern A: “overexertion”

High scores in the dimensions of professional commitment reflect the importance of work for participants with this behavior pattern. Lower scores in the ability to cope with stress and emotional wellbeing show the “costs” of this exhaustive behavior. The label indicates similarities to the concept of type-A behavior that has been described as a risk factor for coronary artery disease and myocardial infarction [26].

#### Risk-pattern B: “burnout”

Low scores in the dimensions of professional commitment and unfavorable scores in the dimensions related to the domain of resistance toward stress characterize this pattern. In addition, we find low scores for satisfaction with work and life as well as for social support. This risk pattern comprises the core symptoms of burnout [35].

The pattern of a study participant is determined by estimating the concurrence of the individual data score and the four reference profiles (weighted linear combination based on an algorithm of discriminant analysis; e.g., probability scores p_G_ = 0.75, p_S_ = 0.15, p_A_ = 0.06, and p_B_ = 0.04 classified as type G).

The instrument has been validated previously by comparing the results with scales that measured related constructs (e.g., Freiburg Personality Inventory (FPI), Maslach Burnout Inventory (MBI), Big-Five Adjective List) and moderate to good correlations were found. Significant differences between the four patterns in terms of psychological symptoms (e.g., highest scores in exhaustion in risk pattern B) and other health related constructs and conditions, support the health relevance of the four behavior and experience patterns [35].

### Data analysis

Data analyses were conducted with SPSS for Windows, Version 15.0 (SPSS Inc., Chicago, IL, USA). Differences of the pattern distribution (categorical variables) were analysed with χ^2^- Test (cross-sectional) and McNemar/Bowker Test (longitudinal) followed by a descriptive analysis of the differences of the single patterns with consideration of standard residuals. Results of categorical analyses were reported as percentages. For continuous variables and longitudinal analyses, we used analyses of variance for repeated measures. The ANOVA models consisted of the within-subject factor time (4 levels: t0, t1, t2, and t3), the between subject factor sex (2 levels: male and female) and the interaction effect of both factors (time x sex). Interaction effects were further analyzed by conducting separate repeated measure ANOVAs for males and females and following t-test for dependent samples to test differences between the levels of the factor time. Results of univariate analyses of this data were reported as means ± standard deviation. Effect size was reported as partial eta square. All analyses were computed for STEM and medical students separately. For the identification of datasets in the longitudinal analyses the participants were asked to generate a personal identification code and/or provide the matriculation number.

## Results

**Fig. 1:**
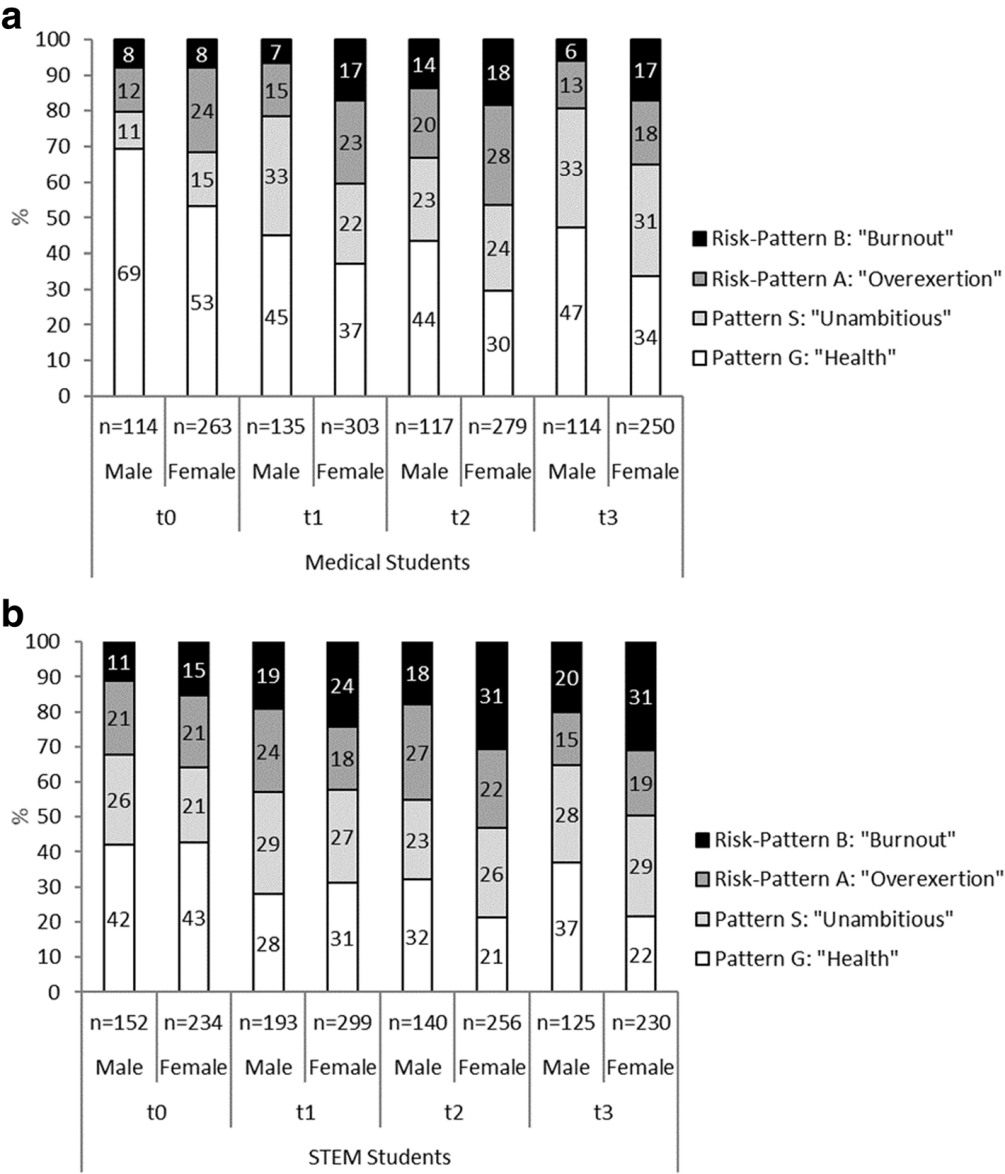
**a** Distribution of study-related behavior and experience patterns of male and female medical students. (p _Male vs Female in medical_; t0 = 0.020, t1 = 0.001, t2 = 0.047, t3 = 0.007; p _Male medical vs STEM_: t0 < 0.001, t1 < 0.001, t2 = 0.218, t3 = 0.013; p _Female medical vs STEM_: t0 = 0.008, t1 = 0.033, t2 = 0.002, t3 = 0.001). **b** Distribution of study-related behavior and experience patterns of male and female STEM students (p _Male vs Female in STEM_: t0 = 0.585, t1 = 0.239, t2 = 0.010, t3 = 0.012)

Each year about 185 medical students start their course of study at the University of Lübeck. The response rate in medical students varied from 65 to 80% (in total *n* = 584 respondents answered for at least one time point; for the number of respondents at each time point see Fig. 1; *n* = 206 answered at all time points). Due to a high proportion of students not showing up after enrolment or quitting during the first year it is not possible to report the corresponding response rate for STEM students (in total *n* = 757 respondents; for the number of respondents at each time point see Fig. 1; *n* = 130 answered at all four time points). Overall, 66.6% of medical students who participated were female (56.7% female STEM students). In both groups there were only minor variations in terms of number of participants and gender distribution across the measuring points (see gender split numbers of participants in Fig. 1). The maximal response was at t1 with a moderate decrease up to t3. Pattern distribution between cohorts 2011, 2012 and 2013 were not significantly different in t0 and t3 but different for Med in t2 and STEM in t1 (in both cases according to standard residuals mainly due to a higher proportion of students with pattern S and lower proportion with risk pattern A in 2012). The mode of age in both groups was 19 (t0), 20 (t1), 21 (t2), and 22 years (t3).

### Behavior and experience patterns

**Fig. 2:**
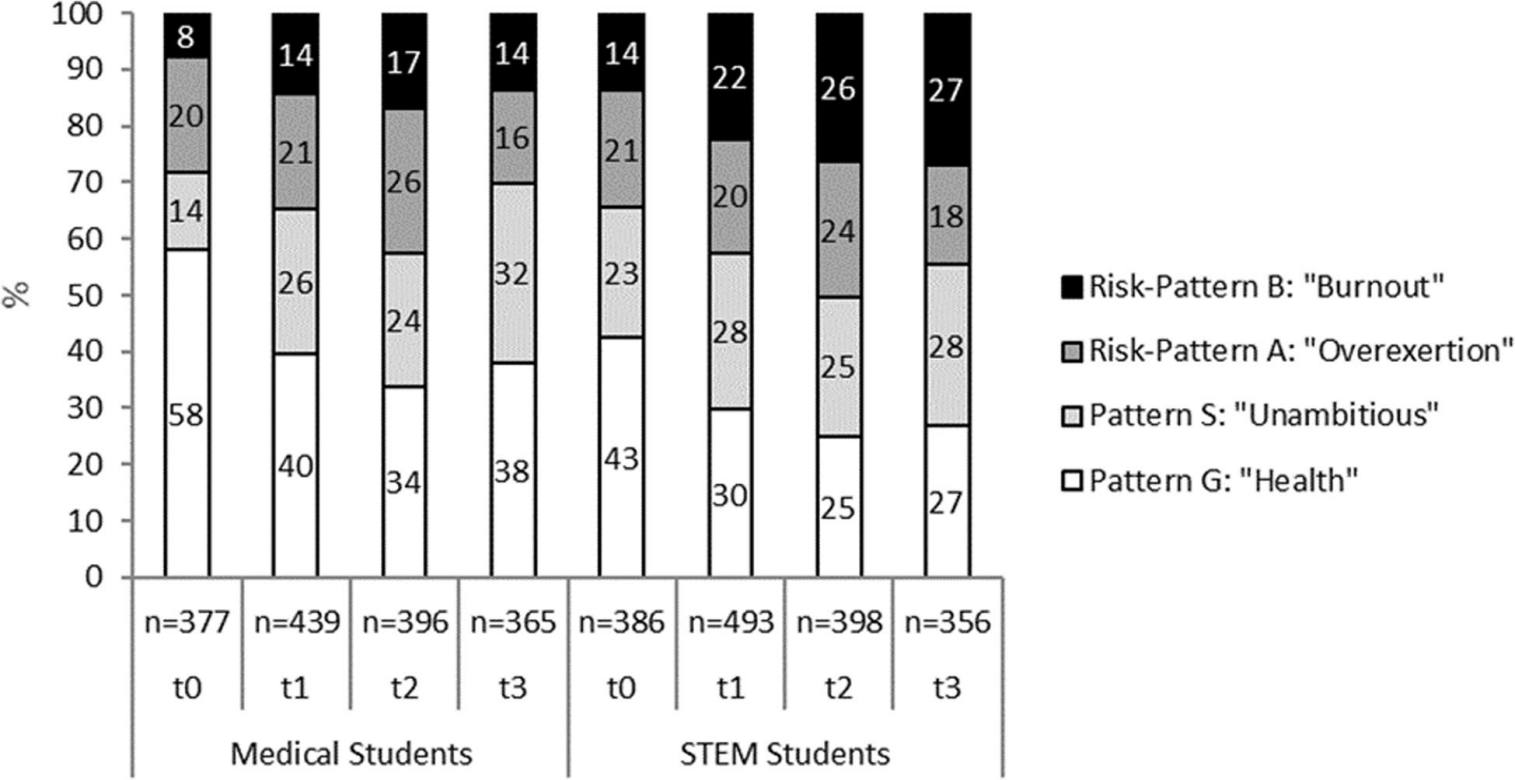
Study-related behavior and experience patterns in medical and STEM students (total groups; statistical differences of medical vs. STEM students: t0 *p* < 0.001, t1 *p* = 0.002, t2 *p* = 0.004, t3 *p* < 0.001)

Figure 2 presents the health-related behavior and experience patterns of all medical and STEM students from t0 to t3 (cross sectional analysis). The distribution of patterns between medical and STEM students was significantly different at each of the four measuring points (cross sectional analysis of significant differences of the pattern distribution with χ^2^-Test followed by a descriptive analysis of the differences of the single patterns with consideration of standard residuals). Freshman medical students presented with a larger proportion of the healthy pattern (58.1%) than STEM students (42.5%). In both groups this proportion decreased to 33.8% (Med)/25.1% (STEM) at t2, with only a minor improvement at t3 (38.1(Med)/27.0% (STEM)). Correspondingly, the proportion with the burnout-related risk pattern B increased from 8.0% (Med)/13.7% (STEM) to 16.9% (Med)/26.4% (STEM) at t2. Again, there was a minor improvement from t2 to t3 in medical students (13.7%) but a continuous increase in STEM students (27.0%). Likewise, there was an increase of pattern S, with reduced study motivation from t0 13.8 (Med)/23.1% (STEM) to t3 31.8 (Med)/28.4% (STEM).

**Fig. 3:**
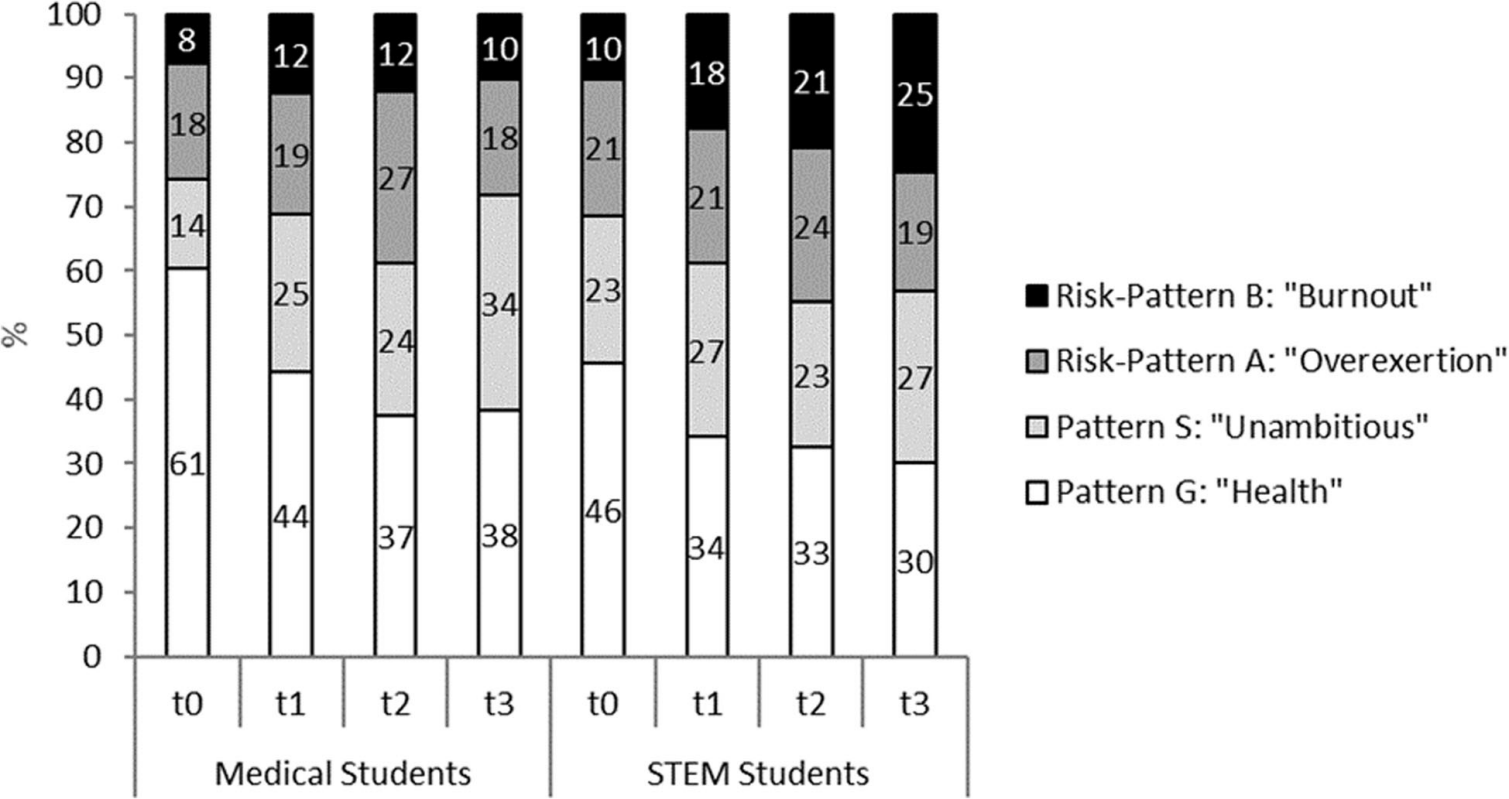
Study-related behavior and experience patterns in medical and STEM students (students who responded at all timepoints (Med *n* = 206; STEM *n* = 130; statistical differences of medical vs. STEM students: t0 *p* = 0.047, t1 *p* = 0.252, t2 *p* = 0.191, t3 *p* = 0.004)

In general, the distribution of patterns in those students who responded at all time points (Med *n* = 206, STEM *n* = 130) longitudinal analysis) was comparable to the cross sectional analysis, but the proportion of students with pattern G was slightly higher and the proportion of students with risk-pattern B slightly lower than in the total group (Fig. 3). The distribution of patterns for those students who answered at all time points was significantly different between medical and STEM students at t0 and t3 (*p* < 0.05).

The distribution of patterns between male and female medical students was significantly different at each of the four measuring points (cross sectional analysis of significant differences of the pattern distribution with χ^2^- Test followed by a descriptive analysis of the differences of the single patterns with consideration of standard residuals). The proportion of female students with the healthy pattern was constantly lower than that of the male students. Both genders started with a small and almost similar proportion of the burnout-related risk pattern but, at the following time points, the proportion of female students was constantly higher (Fig. 1). In STEM students these differences were less pronounced and significant only at t2 and t3. At t0 and t1 the proportion of female STEM students with the healthy pattern was even slightly higher than that of male students, but reversed at t2 and t3. The proportion of female STEM students with the burnout related pattern was constantly higher throughout all measuring points compared to their male counterparts. (Due to the much smaller number of participants, who responded at all time points, further reduced in the analysis by the subdivisions of Med/STEM, male/female and the distribution across four patterns a longitudinal analysis was not possible here).

### Dimensions

Repeated Measures Analysis of Variance with the factors time (within-subject factor), sex (between subject factor) and the interaction term have been computed for medical and for STEM students separately to test for changes in the AVEM dimensions over time. In these analyses only students who responded at all measuring points were included. In both medical and STEM students in 8 of 11 dimensions there was a significant change over time (Tables 1 and 2). The strongest effect sizes were observed in medical students in the decrease in the dimensions career ambition (F (2.8, 565) = 37.8, *p* < 0.001, effect size part. η^2^ = 0.158), striving for perfection (F (2.8, 562) = 30.8, *p* < 0.001, effect size part. η^2^ = 0.134) and the initial decrease and later increase of emotional distancing (F (3, 603) = 29.9, *p* < 0.001, effect size η^2^ = 0.129). In STEM students the strongest effect size was observed in the decrease in satisfaction with life (F (2.7, 344) =13.8, *p* < 0.001, effect size part. η^2^ = 0.096). As with medical students, strong effect sizes were also noted for the decrease in the dimensions career ambition (F (2.8, 359) = 9.7, *p* < 0.001, effect size part. η^2^ = 0.070) and striving for perfection (F (2.5, 328) = 8.4, *p* < 0.001, effect size part. η^2^ = 0.061). In STEM students the effect size for the decrease in the dimension of social support was much higher than in medical students.

**Table 1:** Health-relevant dimensions (AVEM) in male and female. Medical students

	Medical students^a^												p_time_	part. Eta^2^	p_sex_	part. Eta^2^	*P* < 0.05
	t0			t1			t2			t3							
	Total	Male	Female	Total	Male	Female	Total	Male	Female	Total	Male	Female					
	M (SD)	M (SD)	M (SD)	M (SD)	M (SD)	M (SD)	M (SD)	M (SD)	M (SD)	M (SD)	M (SD)	M (SD)					
Subjective significance of work	11.9 (3.0)	11.7 (3.1)	12.0 (3.0)	11.7 (3.0)	11.9 (3.2)	11.6 (2.9)	11.4 (3.2)	11.9 (3.5)	11.3 (3.1)	10.9 (3.4)	11.6 (3.7)	10.6 (3.3)	0.006	0.022	0.356	0.004	0-3^b^, 1–3
Career ambition	15.3 (2.6)	15.2 (3.0)	15.3 (2.4)	13.7 (2.9)	14.5 (3.1)	13.4 (2.8)	13.5 (2.9)	13.9 (3.1)	13.4 (2.9)	13.4 (3.0)	13.8 (3.3)	13.2 (2.9)	<.001	0.158	0.186	0.009	0–1, 0–2, 0–3
Tendency to exert	12.4 (2.9)	11.7 (2.5)	12.6 (3.0)	11.6 (3.0)	11.2 (2.7)	11.7 (3.1)	12.1 (3.2)	11.7 (2.8)	12.3 (3.3)	11.6 (3.1)	11.3 (2.6)	11.7 (3.3)	0.002	0.026	0.134	0.011	0–1, 0–3, 1–2
Striving for perfection	14.8 (2.9)	14.4 (2.8)	14.9 (2.9)	13.4 (2.9)	13.0 (3.2)	13.6 (2.8)	13.1 (3.3)	12.4 (3.1)	13.3 (3.4)	13.2 (3.1)	13.1 (3.1)	13.3 (3.2)	<.001	0.134	0.187	0.009	0–1, 0–2, 0–3
Emotional distancing	12.9 (2.4)	13.3 (2.3)	12.8 (2.4)	12.6 (2.8)	12.6 (2.6)	12.6 (2.9)	11.8 (2.9)	11.8 (3.0)	11.8 (2.9)	13.8 (2.9)	13.9 (3.1)	13.8 (2.9)	<.001	0.129	0.734	0.001	0–2, 0–3, 1–2, 1–3, 2–3
Resignation tendencies	11.8 (2.9)	11.2 (2.7)	12.0 (3.0)	11.7 (2.9)	11.0 (2.9)	11.9 (2.9)	11.6 (3.2)	10.9 (2.9)	11.8 (3.2)	11.3 (3.3)	10.5 (3.2)	11.5 (3.3)	0.034	0.015	0.025	0.025	–
Offensive coping with problems	14.0 (3.0)	14.3 (2.9)	13.9 (3.0)	13.8 (2.9)	13.9 (3.0)	13.8 (2.8)	13.7 (2.9)	14.1 (2.9)	13.6 (2.9)	13.6 (2.9)	14.3 (2.9)	13.4 (2.8)	0.448	0.004	0.192	0.008	–
Balance and mental stability	13.5 (3.3)	14.7 (3.3)	13.0 (3.0)	13.9 (3.0)	15.4 (2.5)	13.3 (3.0)	13.4 (3.2)	14.7 (2.8)	12.9 (3.2)	13.7 (3.1)	14.7 (2.9)	13.3 (3.1)	0.017	0.017	<.001	0.083	0–1, 1–2
Satisfaction with work	17.4 (2.4)	17.2 (2.5)	17.6 (2.4)	17.1 (2.5)	17.1 (2.3)	17.2 (2.5)	17.1 (2.5)	16.8 (2.5)	17.2 (2.5)	17.0 (2.5)	17.2 (2.2)	17.0 (2.6)	0.261	0.007	0.603	0.001	–
Satisfaction with life	17.6 (2.5)	17.7 (2.6)	17.5 (2.5)	17.1 (2.6)	17.3 (2.3)	17.0 (2.7)	16.8 (2.8)	16.9 (2.9)	16.7 (2.7)	17.2 (2.6)	17.3 (2.6)	17.1 (2.6)	<.001	0.032	0.536	0.002	0–1, 0–2
Experience of social support	18.0 (2.0)	18.0 (2.1)	18.0 (2.0)	17.7 (2.1)	17.9 (2.3)	17.7 (2.1)	17.4 (2.5)	17.5 (2.4)	17.4 (2.6)	17.3 (2.6)	17.3 (2.5)	17.4 (2.6)	0.001	0.027	0.946	<.001	0–2, 0–3

^a^In this analysis same number of participants for t0 to t3: Total *n* = 204, Male *n* = 55, Female *n* = 149

^b^Indicates a significant difference of total group scores between t0 and t3

**Table 2:** Health-relevant dimensions (AVEM) in male and female. STEM students

	STEM Students^a^												p_time_	part. Eta^2^	p_sex_	part. Eta^2^	*P* < 0.05
	t0			t1			t2			t3							
	Total	Male	Female	Total	Male	Female	Total	Male	Female	Total	Male	Female					
	M (SD)	M (SD)	M (SD)	M (SD)	M (SD)	M (SD)	M (SD)	M (SD)	M (SD)	M (SD)	M (SD)	M (SD)					
Subjective significance of work	11.8 (2.9)	11.8 (3.1)	11.8 (2.8)	11.8 (3.0)	11.8 (3.5)	11.8 (2.8)	11.8 (3.2)	(12.1 (3.5)	11.7 (3.1)	11.2 (2.9)	11.3 (3.1)	11.1 (2.8)	0.029	0.023	0.698	0.001	2-3^b^
Career ambition	14.9 (2.7)	14.4 (2.9)	15.0 (2.6)	14.3 (2.6)	13.7 (2.9)	14.6 (2.4)	13.9 (2.5)	13.7 (2.7)	14.0 (2.5)	13.7 (2.7)	13.6 (2.7)	13.7 (2.7)	<.001	0.070	0.259	0.01	0–1, 0–2, 0–3
Tendency to exert	11.5 (3.2)	11.3 (3.4)	11.6 (3.2)	11.6 (3.2)	11.3 (3.1)	11.7 (3.3)	11.8 (3.5)	11.8 (3.5)	11.8 (3.5)	11.8 (3.3)	12.1 (3.4)	11.7 (3.3)	0.332	0.009	0.871	<.001	–
Striving for perfection	14.5 (3.0)	14.1 (3.6)	14.6 (2.7)	13.2 (3.4)	13.8 (3.4)	13.0 (3.3)	13.3 (3.6)	13.3 (3.4)	13.3 (3.7)	13.8 (3.2)	13.9 (3.2)	13.8 (3.2)	<.001	0.061	0.821	<.001	0–1, 0–2
Emotional distancing	12.9 (2.8)	13.0 (2.8)	12.9 (2.8)	12.6 (3.0)	12.0 (2.9)	12.9 (3.0)	12.4 (3.1)	12.2 (2.5)	12.5 (3.4)	12.8 (3.2)	11.9 (3.1)	13.2 (3.3)	0.168	0.013	0.207	0.012	–
Resignation tendencies	11.6 (3.1)	11.0 (3.0)	11.8 (3.1)	11.8 (3.1)	10.8 (3.0)	12.3 (3.0)	11.8 (3.3)	10.4 (2.8)	12.5 (3.4)	12.0 (3.2)	11.0 (3.2)	12.5 (3.1)	0.552	0.005	0.003	0.067	–
Offensive coping with problems	13.3 (2.5)	13.9 (2.4)	13.0 (2.5)	12.5 (2.9)	13.2 (3.0)	12.2 (2.9)	12.4 (3.0)	13.2 (3.4)	12.0 (2.8)	12.3 (2.6)	13.1 (2.8)	11.9 (2.5)	<.001	0.048	0.016	0.045	0–1, 0–2, 0–3
Balance and mental stability	13.2 (3.2)	13.4 (3.5)	13.0 (3.1)	13.8 (2.9)	14.4 (3.0)	13.5 (2.9)	13.2 (3.1)	13.6 (3.1)	13.0 (3.1)	12.9 (3.3)	13.4 (3.4)	12.7 (3.3)	0.002	0.039	0.216	0.012	0–1, 1–2, 1–3
Satisfaction with work	16.3 (2.5)	16.1 (2.6)	16.4 (2.4)	15.8 (2.7)	16.0 (2.9)	15.7 (2.6)	15.7 (2.8)	16.0 (2.7)	15.6 (2.8)	15.6 (3.0)	15.1 (2.8)	15.9 (3.0)	0.01	0.031	0.850	<.001	0–3
Satisfaction with life	16.7 (2.7)	16.3 (2.9)	16.8 (2.5)	15.9 (2.9)	15.4 (3.1)	16.1 (2.9)	15.5 (3.1)	15.3 (2.9)	15.6 (3.1)	15.4 (3.4)	14.6 (3.1)	15.8 (3.5)	<.001	0.096	0.183	0.014	0–1, 0–2, 0–3
Experience of social support	18.1 (1.9)	17.8 (1.7)	18.3 (2.0)	17.9 (2.4)	17.4 (2.6)	18.2 (2.3)	17.2 (2.9)	17.1 (2.8)	17.2 (3.0)	17.0 (2.9)	16.4 (2.6)	17.2 (3.1)	<.001	0.071	0.168	0.015	0–2, 0–3, 1–3

^a^In this analysis same number of participants for t0 to t3: Total *n* = 131, Male *n* = 41, Female *n* = 90

^b^Indicates a significant difference of total group scores between t2 and t3

Both medical and STEM female students had significantly higher scores in resignation tendencies (*p* = 0.025 Med; *p* = 0.003 STEM) compared with their male colleagues. Male medical students had higher scores for balance and mental stability (*p* < 0.001), and STEM students in offensive coping with problems (*p* = 0.016), compared with their female colleagues (Tables 1 and 2). Furthermore, time effects were qualified by sex for the dimensions subjective significance of work, career ambition, and satisfaction with work for medical students and for the dimension satisfaction with work in STEM students. These interaction effects reveal a decrease of values which is overall more pronounced for females than for males.

## Discussion

As one of the rare longitudinal studies not addressing just one study group, this survey examines the development of the study-related behavior of medical students in comparison with STEM students at one German university. In STEM students we found a substantial and increasing fraction at risk for burnout, and a decreasing fraction with a healthy study-related behavior and experience pattern. In Med students this was true up to t2. Female students showed a more unfavorable distribution of patterns and a higher vulnerability, especially in the area of resistance toward stress.

### Development of behavior and experience patterns

At the beginning of the course of study most of these medical students presented with a healthy behavior and experience pattern. However, consistent with our first hypothesis, the proportion with this pattern continually diminished, with only a slight recovery at t3. Correspondingly, the proportion of students with a pattern at risk for burnout increased, again with a small recovery at t3. The slight recovery is consistent with passing the first important examination after the second year. However, with 20% less students with a healthy pattern compared to t0 study strain is still highly visible. These results confirm reports about the increasing perception of psychosocial strain in the course of medical studies [5, 8, 32]. However, given the results of the STEM students, this does not seem to be exclusive for medical education. Starting with a lower proportion with the healthy pattern and a higher proportion of the burnout-related pattern, in these students the same decrease in the proportion of students with the healthy pattern as well as the increase in students with the burnout-related pattern was found. At t2 and t3 STEM students had about ten percentage points higher proportions at risk for burnout than medical students. The fraction with the healthy pattern was about the same amount smaller in STEM students (t2 25%, t3 27%) compared to medical students (t2 34%, t3 38%). However, there was no recovery regarding the burnout related risk-pattern B at t3 in STEM students. This difference might be explained by the fact that the third year for BA students is their final year before graduation, while for medical students it is the year after the first extensive examination.

### Stress not particularly pronounced in medical students

Given the same direction of changes in the distribution of patterns but significantly differing percentages between medical and STEM students, our second hypothesis was only partly confirmed. Results from other studies support that stress may not be particularly pronounced in medical education. Levels of distress were lower in medical students compared to law or business students [17]. Prevalence of depression was lower or similar to other student groups [9]. US medical students started with better health compared to age-matched college students at matriculation [16]. However, they had poorer psychosocial health over the course of study [2]. Medical students spent significantly more hours studying than law students [15] but a comparable amount of time required for studying was also stated for chemistry students [38]. While high stress levels in medical education have constantly been reported [39] the lack of comparison studies may have concealed that students of other subject may perceive comparable stress. At least since the implementation of BA and MA programs in Germany, increasing stress of students in the affected subjects has been reported [18]. The fraction of students who worried about whether they would be able to graduate successfully increased. Most particularly, the strictly-scheduled course of study, including a high workload and frequent testing, was claimed by students to cause stress [38]. More than a quarter of STEM students at risk for burnout, and another quarter with reduced study motivation at the time of graduation in our survey, supports this impression of perceived stress. Given the small numbers of studies that compare not only students of different study subjects but also with age matched peers, the question arises whether the reported distress of single student groups may just be a function of age and adolescence. However, according to a study at the University of Adelaide the proportion of distressed students was 4.4 times higher than in age matched peers [17]. In summary, we conclude that the results of our study reflect a comparable psychosocial strain at an elevated level in both student groups. Since there is no training for medical students to deal with this stress adequately nor systematic efforts to reduce workload or optimize teaching conditions this might not be a good starting base for an even more demanding professional work-life. Reports about higher anxiety, depression, burnout and suicide levels of physicians compared to the general population or professions with a comparable responsibility support this point of view [40–43]. However, resilience training is not common for STEM students (or other study subjects), either. Increasing sick leave time due to mental health problems over the last years in the general working population in Germany demonstrates - as with students - a tendency for other professionals in the same direction as physicians regarding psychosocial strain.

### Development of dimensions

The changes in the distribution of patterns reflect the unfavorable changes in all three areas of the AVEM dimensions, with strongest effect sizes in both groups in study-related commitment. Of note in both groups is also the significant decrease in the dimensions of satisfaction with life and social support. Other studies have shown that neglecting social networks due to high workload is a typical coping mechanism of medical students [44, 45].

According to our results the STEM students are affected likewise with an even stronger effect size than in medical students. However, in both groups the total values do not support the perception of a critical level of social isolation. The decreasing tendency should nevertheless be observed carefully, since social support has been found to be one of the most effective resources in the prevention of burnout [46, 47].

### Gender differences in the experience of stress and coping resources

As put forth in our third hypothesis, in both student groups female students showed a more unfavorable distribution of patterns with a lower proportion of the healthy pattern (all time points in medical students, t2 and t3 in STEM students) and a higher proportion of the burnout-related pattern than their male fellow students. In both groups female students were found to have a significantly higher vulnerability, especially in the domain of resistance toward stress, which was seen in higher tendencies towards resignation (both groups), and lower scores in balance and mental stability (medical students), as well as in offensive coping with problems (STEM students). In three of eleven dimensions in medical students (subjective significance of work, career ambition, and satisfaction with work) and one dimension in STEM students (satisfaction with work) there was a stronger decrease over time in female compared to male students.

A different perception of and reaction to study-related stress in female students has been reported by a number of other studies. A review of psychosocial distress in medical students reported a higher risk for female students [48]. In a representative survey of German students, 54% of female students, compared to only 35% of male students, felt exhausted by study-related stress [49]. Higher proportions of female students were stressed by examinations, heavy workload, and the fear of poor grades. To a higher degree they also showed symptoms that could be perceived as psychosomatic reactions to study-related stress, such as headache, back pain, sleep disturbance, stomach pain, difficulties in concentration or frequent colds. Besides higher levels of anxiety, being female was also one of the strongest predictors for participating in a progressive muscle-relaxation intervention for stress reduction in medical students [50]. Since the majority of medical students in Germany are female, and their numbers in STEM education are increasing, these different perceptions and reactions to study-related stress have to be emphasized in study organization, teaching, and preventive and health-promoting strategies. A program of Progressive Muscle Relaxation significantly reduced anxiety and increased the quality of life in female students [51].

### Strengths and limitations

A strength of our study is that it is one of the rare long-term longitudinal studies that compare different student groups. We analyzed both longitudinal and cross-sectional data, and there was good alignment between these data (see Figs. 2 and 3). Limitations include that the study is based on a self-report instrument and that the response rate of medical students was better than the estimated response rate of STEM students, so a selection bias for the latter could not be completely ruled out. Similarly, a higher percentage of female medical students participated in the study than the estimated percentage of female STEM students. Due to students who are enrolled but do not show up, as well as quite a number of students that quit early, we were not able to name the exact ratio of respondents vs. total group of active STEM students. The study was conducted in one German university, and so the results should not be generalized for all German medical and STEM students or to other nations.

### Implications for health promotion and future research

It has been shown that risk patterns were associated with symptoms of stress and mental illness [25]. It has also been proven that students who experience stress perform poorer in tests and examinations [52, 53]. The wealth of information to be learnt and frequent testing also leads to a student attitude that is focused on learning to pass the exams and much less on the joy of studying subjects of interest. Therefore, it has to be in the interest of universities to revise the course of study and to support students with coping strategies to change these development patterns. Special emphasis has to be placed on addressing the specific needs of female students. At the level of individual behavior, courses in relaxation techniques like Autogenic Training or Progressive Muscle Relaxation as well as mindfulness-based meditation practices have been proven to decrease stress levels and symptoms of anxiety and depression in medical students [51, 54, 55]. Even more important would be more comprehensive programs at university level that integrate behavior and context related measures. Such programs are rare but show promising results [56, 57]. A number of reform programs have been established for medical education in Germany. In contrast to the focus on natural sciences in the explicitly labeled pre-clinical first 2 years of the standard curriculum, central elements in the reform courses are the early integration of clinical information and patient contact and changes in student examinations [58]. Usually this improves study satisfaction. They might also help to reduce stress and depression and enhance self-efficacy [59]. However, self-reflective elements and training of stress management and coping are neither essential parts of the standard nor on the reform programs. After years of political discussion in 2015 a Prevention Act was passed in Germany that enables the statutory health insurance to fund preventive and health promoting measures and programs. A central target is settings, explicitly including educational environments. In reviving the Healthy University Programs as part of the setting approach of the Ottawa Charter of the 1980s and funded by health insurances, a number of universities in Germany are developing programs for health promotion for students and employees with promising results [60]. Further analysis of the ongoing longitudinal study will show if the slight alleviation in the distribution of pattern G and risk pattern B in medical students (and of pattern G in STEM students) will continue. An important issue for future research would also be to conduct multicentered studies in order to deliver generalizable results about student stress and successful coping across study subjects. Since the AVEM measures the interplay between the experience of stress and the individual’s coping behavior, it would be of interest for future research to measure stress directly and correlate the results with the AVEM patterns.

## Conclusions

The study set out to compare the development of psychosocial stress in medical and STEM students at one German university. Given that there has been much research into and comment about the impact of medical education on the mental health of the former, within the context of the duration of this study medical students of did not appear to be more negatively affected than their STEM counterparts. The substantial and increasing fractions of students at risk for burnout, and the decreasing fractions with a healthy study-related behavior and experience pattern, may emphasize the need for prevention and health promotion on an individual and a contextual level for both of these student groups. This is likely to improve students’ health and performance.

## Data Availability

The datasets used and/or analysed during the current study are available from the corresponding author on reasonable request.

## Abbreviations

AVEM: “Work-Related Behavior and Experience Pattern” [Arbeitsbezogenes Verhaltens- und Erlebensmuster]
STEM: Students of Science, Technology, Engineering and Mathematics
